# Publisher preferences for a journal transparency tool: A modified three-round Delphi study

**DOI:** 10.12688/f1000research.154408.1

**Published:** 2024-08-09

**Authors:** Jeremy Y. Ng, Henry Liu, Mehvish Masood, Rubaina Farin, Mireille Messih, Amaya Perez, IJsbrand Jan Aalbersberg, Juan Alperin, Gregory L. Bryson, Qiuxia Chen, Alan Ehrlich, Alfonso Iorio, Wim J. N. Meester, John Willinsky, Agnes Grudniewicz, Erik Cobo, Imogen Cranston, Phaedra Eve Cress, Julia Gunn, R. Brian Haynes, Bibi Sumera Keenoo, Ana Marušić, Eleanor-Rose Papas, Alan Purvis, João de Deus Barreto Segundo, Pathiyil Ravi Shankar, Pavel Stoev, Josephine Weisflog, Margaret Winker, Kelly D. Cobey, David Moher

**Affiliations:** 1 Centre for Journalology, Clinical Epidemiology Program, Ottawa Hospital Research Institute, Ottawa, Ontario, Canada; 2 Department of Health Research Methods, Evidence, and Impact, Faculty of Health Sciences, McMaster University, Hamilton, Ontario, Canada; 3 Elsevier, Amsterdam, The Netherlands; 4 School of Publishing, Simon Fraser University, Vancouver, British Columbia, Canada; 5 Department of Anesthesiology and Pain Medicine, University of Ottawa, Ottawa, Ontario, Canada; 6 EBSCO Information Services, Ipswich, Massachusetts, USA; 7 Department of Family Medicine and Community Health, UMass Chan Medical School, Worcester, Massachusetts, USA; 8 Department of Medicine, McMaster University, Hamilton, Ontario, Canada; 9 Graduate School of Education, Stanford University, Stanford, California, USA; 10 Telfer School of Management, University of Ottawa, Ottawa, Ontario, Canada; 11 Department of Statistics and Operations Research, Barcelona-Tech, UPC, Barcelona, Spain; 12 F1000, Taylor & Francis, London, UK; 13 The Aesthetic Society, Garden Grove, California, USA; 14 Taylor & Francis, London, UK; 15 Faculty of Medicine and Health Sciences, University of Mauritius, Réduit, Moka, Mauritius; 16 Department of Research in Biomedicine and Health, Center for Evidence-based Medicine, University of Split School of Medicine, Split, Croatia; 17 Independent Researcher, Croydon, London, UK; 18 Hong Kong Academy of Medicine Press, Hong Kong, Hong Kong; 19 Escola Bahiana de Medicina e Saude Publica, Salvador, State of Bahia, Brazil; 20 IMU Centre for Education, IMU University, Kuala Lumpur, Malaysia; 21 National Museum of Natural History at the Bulgarian Academy of Sciences, Sofia, Bulgaria; 22 Pensoft Publishers, Sofia, Bulgaria; 23 BMJ Publishing Group, London, UK; 24 Program Director and Trustee, World Association of Medical Editors, Bellagio, Italy; 25 School of Epidemiology and Public Health, Faculty of Medicine, University of Ottawa, Ottawa, Ontario, Canada; 26 University of Ottawa Heart Institute, Ottawa, Ontario, Canada

**Keywords:** health literacy, scholarly publishing, periodicals as topic, publications, ethics in publishing, journal transparency tool

## Abstract

**Background:**

We propose the creation of a journal transparency tool (JTT), which will allow users to obtain information about a given scholarly journal’s operations and policies. We are obtaining preferences from different stakeholders to inform the development of this tool. This study aimed to identify the publishing community’s preferences for the JTT.

**Methods:**

We conducted a modified three-round Delphi survey. Representatives from publishing houses and journal publishers were recruited through purposeful and snowball sampling. The first two Delphi rounds involved an online survey with items about JTT metrics and user features. During the third round, participants discussed and voted on JTT metric items that did not reach consensus after round 2 within a virtual consensus meeting. We defined consensus as 80% agreement to include or exclude an item in the JTT.

**Results:**

Eighty-six participants completed the round 1 survey, and 43 participants (50% of round 1) completed the round 2 survey. In both rounds, respondents voted on JTT user feature and JTT metric item preferences and answered open-ended survey questions regarding the JTT. In round 3, a total of 21 participants discussed and voted on JTT metric items that did not reach consensus after round 2 during an online consensus group meeting. Fifteen out of 30 JTT metric items and none of the four JTT user feature items reached the 80% consensus threshold after all rounds of voting. Analysis of the round 3 online consensus group transcript resulted in two themes: ‘factors impacting support for JTT metrics’ and ‘suggestions for user clarity.’

**Conclusions:**

Participants suggested that the publishing community’s primary concerns for a JTT are to ensure that the tool is relevant, user-friendly, accessible, and equitable. The outcomes of this research will contribute to developing and refining the tool in accordance with publishing preferences.

## Introduction

Peer-reviewed scholarly journals play a critical role in disseminating biomedical research. Scientific publications must be reported and disseminated according to best-practice standards to be of use to clinicians, researchers, patients, and others. For example, published research should be properly indexed and archived so that it can be found and preserved over time. Several calls have been made for academic journals to become more transparent, including initiatives to open the peer-review process and to make study data publicly available. ^1^^,^^2^ This project is part of a program of research being conducted to establish user needs and preferences for a journal transparency tool (JTT) within the biomedical sciences. A JTT, as we envision it, could be an automated tool to provide users with information about a given journal’s transparency practices. This in turn could help users decide on the journal’s credibility and whether to interact with it (e.g., read and cite articles published in it, submit manuscripts to it). The tool is expected to add value by providing stakeholders with information about how a journal operates and which best practices it meets. The benefit of this tool may be most obvious for producers of research looking to publish, as well as readers of research looking to consume evidence to help inform healthcare-related decisions. Additionally, this tool may be of great value to new researchers that are being introduced to the research community and individuals in the profession of evaluating, publishing, and indexing research.

We have identified three types of stakeholders whose preferences would be important to consider when developing the JTT: (1) publishers; (2) researchers and clinicians ^3^; and (3) patients. ^4^ Here, we focus on the needs and preferences of group 1, namely those working in the publishing industry, including full-text publishers, discovery services (e.g., Web of Science, Scopus, PubMed), preprint servers, self-published journals, university-published journals, and reference managers. Publishers are intimately involved in the dissemination of research. Publishing houses have a duty to support the journals that operate under their umbrella. In contrast, editors have responsibility for manuscript evaluation, including peer review, revisions, selection of manuscripts for publication, and editorial content. For the purposes of this analysis, editors are included under the umbrella term of ‘publishers.’ The aim of this study was to identify preferences for a JTT within the international publisher community. This study is descriptive, and we had no *a priori* hypotheses.

We conducted a modified three-round Delphi survey with a synchronous online consensus exercise to determine publishing community preferences. The Delphi method is a means of structuring communication between experts around a complex issue to make a collective judgment on a given topic. It is a social science technique but has been applied in other settings, including healthcare environments (see Refs. 5, 6 for examples). The Delphi approach allows for effective communication by individual group members and the group as a whole. ^7^ It limits direct confrontation between individuals, fosters independent thought by the experts involved, and aims to achieve consensus within a group without the need for a leader. ^8^ By using the Delphi method, we established the international English-speaking publisher communities’ consensus view on what should be included in a JTT, which will contribute to meaningfully situating the tool within the scholarly landscape and help to ensure that the most relevant inputs are used to build the tool.

## Methods

### Research ethics approval and transparency practices

We obtained research ethics board approval for the proposed study from the Ottawa Health Science Network Research Ethics Board (REB ID# 20220132) on April 06, 2022. The final protocol was registered on April 28, 2022, using the Open Science Framework (OSF) ^9^ at https://osf.io/fsmzc. Anonymized voting data and participant responses from rounds 1 and 2, along with the round 3 thematic analysis, were shared publicly using OSF at https://osf.io/8v63x/. We followed the ACcurate COnsensus Reporting Document (ACCORD) checklist in reporting our findings. ^10^

### Study design

We conducted a modified three-round Delphi study, similar to the approach used in a previously published study. ^11^ Diamond et al. have proposed key methodologic criteria to report in publications of Delphi studies, ^12^ which were used to inform the present study. The first two rounds of the Delphi involved completing an online independent survey using SurveyLet. ^13^ The third round consisted of a virtual consensus group meeting with participants from earlier rounds.

### Recruitment of participants

Participants were recruited from a diverse group of academic publishing houses. Purposeful sampling based on this list of potential participants and snowball sampling were used to increase representation and target outreach/invitations. To be eligible, participants needed to be fluent in English. Representatives were invited from publishing houses to participate in our Delphi study using a study advertisement and standardized recruitment script (see https://osf.io/6r3np). Members of the public, patients, or carers were not invited for recruitment in any of the three rounds. Participants were not provided financial compensation for taking part in the study, but those who agreed to participate in the third round –that is, the virtual consensus group meeting– were invited to co-author the present research report.

Our advertisement and recruitment script (see https://osf.io/6r3np) were disseminated (IJA, AM, MW) to the publisher community via two large publishing societies: the Open Access Scholarly Publishing Association (OASPA) ^14^ and the International Association of Scientific, Technical, and Medical Publishers (STM), ^15^ as well as the World Association of Medical Editors (WAME) ( https://www.wame.org/). OASPA provides an international community for open-access publishing and seeks to advance open-access publishing solutions. STM is also global in reach, with more than 140 members, which publish nearly 66% of all journal articles and work to advance trusted research worldwide. WAME is an international, nonprofit voluntary association of editors of peer-reviewed medical journals that seeks to foster international cooperation among and the education of medical journal editors. OASPA, STM, and WAME assisted with recruitment by targeting and sharing the invitation via email with their members. In addition, a member of the research team (JYN) extracted all journals catalogued in Directory of Open Access Journals (DOAJ) and used the RAND function ^16^ on Microsoft Excel to select a random sample of 750 journals. Publishers of these selected journals, which were manually checked to ensure they met the eligibility criteria (RF, MMessih, AP), were also sent our advertisement and recruitment script for participation in the study. Individuals interested in participating were redirected from the invitation to an online survey where they were provided with more information about the study, including participant inclusion criteria, and asked to complete an online informed consent form prior to proceeding.

**Round 1**

Participants were asked to complete an online survey (see https://osf.io/6r3np), which asked about their needs and preferences for a JTT. This survey was purpose-built for the study and included 30 items that addressed the following: (1) participant demographic characteristics (e.g., employment information, years working in the publishing industry, and areas of expertise); (2) JTT user features (i.e., how the JTT user interface should look, how data automation can facilitate the metrics the JTT reports, and how to disseminate a completed JTT to the community and track its uptake); and (3) JTT metrics (i.e., metrics that users will access about each *individual* journal on the JTT to make informed decisions regarding the use of that journal for clinical or research purposes). These survey items were informed and chosen by a team of experts in journalology (JYN, KDC, DM). Survey items regarding JTT metrics were posed in a question format, with responses provided on a 9-point Likert scale ^17^ with endpoints labeled ‘strongly agree’ and ‘strongly disagree.’ Participants were provided with a textbox to provide any comments on each item. Respondents also had the opportunity to suggest additional items for the JTT for participants to consider in round 2 of the survey. This survey was pilot tested by a group of researchers who were not part of the study prior to administration.

Participants were sent the survey on May 23, 2022, followed by two reminder emails, each spaced 1 week apart. Responses to the round 1 Delphi survey were collated, with all information presented in aggregate. For items assessing publishers’ needs and preferences for a JTT, we defined consensus on an item as 80% agreement to include or exclude an item. This cutoff was selected based on findings from a systematic review of Delphi studies. ^12^ In our design, all items with 80% of responses in the top third (7-9) or bottom third (1-3) of our 9-point scale were considered to have reached consensus for inclusion or exclusion. Once items met consensus, they were not included in subsequent rounds. Furthermore, open-ended survey responses from round 1 were analyzed to provide feedback and incorporate any new suggestions for round 2 participants.

**Round 2**

Participants who completed round 1 of the survey were emailed an invitation and link to the complete round 2 of the Delphi survey. Members of the research team (JYN, HL, DM, KDC) created round 2 based on the responses obtained in round 1. It contained all survey items in round 1 that did not reach consensus, as well as any new items or clarifications required based on comments participants made in round 1. Comments provided by individual participants to explain responses to items from the round 1 survey were presented back to all participants for consideration as they re-voted on each item. Participants were also provided the opportunity to suggest new JTT items for respondents in Round 3 to vote on.

Participants were provided with the percentage of scores on each third of the scale for each item not in consensus from round 1, along with a collated and de-identified version of any comments made on each item. Participants were sent the survey on March 6, 2023, followed by two reminder emails, each spaced 1 week apart. We processed responses resulting from round 2 and defined consensus in the same way as in round 1.

**Round 3**

We invited all respondents from round 1 to take part in an online consensus group meeting. If unavailable, an option was provided to participants to send another representative from their publishing organization to take part in round 3 in their place. Participants met virtually on Zoom on December 6, 2023, to establish consensus on any remaining JTT metric items from rounds 1 and 2 of the Delphi. The virtual consensus meeting was moderated by a steering committee with an expertise in journalology (JYN, JA, GLB, QC, WJNM, JW, KDC, DM) to ensure that participants could express their views on any outstanding items while ensuring that the focus of the meeting and goals of final consensus on items were met.

We provided all participants with a summary of the results obtained in round 2 approximately one week prior to the meeting. This summary listed all the JTT metrics that did and did not reach consensus. Items that did not reach consensus were presented with the number and percentage of round 2 participants that identified that specific JTT metric as ‘unimportant’ (1-3 points), ‘neutral’ (4-6 points), and ‘important’ (7-9 points) for inclusion on the tool on a 9-point scale. Participants voted through the anonymous polling feature on the Zoom platform, where they were provided three options to select from during the voting of each item: (1) support for inclusion on the JTT, (2) support for exclusion on the JTT, or (3) abstain from voting. The group’s voting results were presented to participants immediately following receipt of all responses. Members of the steering committee did not vote during round 3, and a separate research member (HL) took field notes during the session.

### Analysis

We report the number of participants that completed each round of the Delphi and their basic demographic characteristics using frequencies and percentages. Data for each round of the Delphi was analyzed successively and presented independently. Quantitative items are presented using frequencies and percentages. For each survey item assessing needs and preferences for the JTT, we indicated whether consensus was reached and, if so, at what round.

Open-question survey responses from rounds 1 and 2 were analyzed using a thematic content analysis. ^18^ Two members of the research team (JYN, HL) aggregated and independently coded responses. Themes were confirmed through multiple iterative meetings between research members (JYN, HL), with all items reaching consensus through discussion. Similarly, for the round 3 online consensus group, an automated Zoom transcript, which was reviewed for accuracy (MMasood), was used to conduct a thematic content analysis. ^18^ One member of the research team (MMasood) coded, combined, and uploaded online consensus group notes into Microsoft Excel and established themes based on the codes. Two members of the research team (JYN, MMasood) subsequently discussed the generated themes to confirm their accuracy and relevance.

## Results

### Deviations from the protocol

Challenges with gaining a sufficient participant sample size led to two deviations from the protocol. First, within round 1, additional participants were recruited from a random sample of 750 journals extracted from DOAJ. Similarly, to increase the sample size, participants in round 3 were recruited from *all* respondents from round 1, rather than from a random, stratified sample of respondents (see Table 1 for participant demographic characteristics).

**Table 1.** Participant characteristics.

Demographic	Participant characteristics	Responses (n, %)	
		Round 1	Round 2
Length of time working in publishing industry	<1 year	1 (1.2%)	1 (2.3%)
	1-3 years	10 (11.6%)	3 (7.0%)
	4-10 years	34 (39.5%)	13 (30.2%)
	11-20 years	21 (24.4%)	12 (27.9%)
	20 years or longer	20 (23.3%)	14 (32.6%)
		N=86	N=43
Personal Role in Publishing	Editor/Editorial Tasks	47 (55.3%)	26 (60.5%)
	Publisher/Publishing Tasks	18 (21.2%)	7 (16.3%)
	Research Integrity/Ethics	6 (7.1%)	3 (7.0%)
	Sales/Marketing	3 (3.5%)	0 (0.0%)
	Other	11 (12.9%)	7 (16.3%)
	Infrastructure	27 (30.7%)	14 (32.6%)
		N=85	N=43
Expertise Related to Publishing (Select all that Apply)	Open access	64 (72.7%)	34 (79.1%)
	Open science	34 (38.6%)	19 (44.2%)
	Publication process	68 (77.3%)	35 (81.4%)
	Partnership	23 (26.1%)	13 (30.2%)
	Integrity	38 (43.2%)	20 (46.5%)
	Learning and education	30 (34.1%)	14 (32.6%)
	Technology	23 (26.1%)	14 (32.6%)
	Content structuring	22 (25%)	10 (23.3%)
	Discovery services	7 (8.0%)	7 (16.3%)
	Application development	12 (13.6%)	10 (23.3%)
	Editor relations	54 (61.4%)	29 (67.4%)
	Editorial processes/Peer review	72 (81.8%)	38 (88.4%)
	Marketing and communications	22 (25.0%)	16 (37.2%)
	Other	7 (8.0%)	3 (7.0%)

Time and resource constraints also resulted in additional changes to the round 3 online consensus group. First, round 3 was conducted within a single day as opposed to two half-days to accommodate participant availability. Second, JTT user features (i.e., preferences for a website or API interface, fee-for-usage, the use of automation, and registration for access on the tool; see Table 2) and JTT metric items (see Table 3) that did not reach consensus in round 2, along with novel JTT metrics suggested by round 2 participants, were originally intended to be discussed and voted in round 3. Instead, *only* JTT metrics items that did not reach consensus in round 2 (see Table 3 for items) were discussed and voted on within round 3.

**Table 2.** Journal Transparency Tool (JTT) user format preferences voting results.

#	Item	Scale	Responses (n, %)	
			Round 1	Round 2
1	Please indicate your preference for whether the JTT should be designed and hosted on a website or be designed and downloadable as a browser plugin or as an API. Select all that apply.	I prefer the tool to be designed/hosted on a website	51 (58.0%)	33 (76.7%)
		I prefer the tool to be designed/downloadable as a browser plugin or API	31 (35.2%)	15 (34.9%)
		I do not feel that I have enough expertise in this area	14 (15.9%)	4 (9.3%)
			N=94	N=43
2	Please indicate your preference for whether the JTT should be fully automated.	Very Strongly Agree, Strongly Agree, or Agree	41 (48.2%)	24 (55.8%)
		Somewhat Agree, No Preference, or Somewhat Disagree	34 (40%)	14 (32.6%)
		Very Strongly Disagree, Strongly Disagree, or Disagree	10 (11.8%)	5 (11.6%)
			N=85	N=43
3	Should users of the JTT have to register to create an account, or should the tool be available without registration?	Very Strongly Agree, Strongly Agree, or Agree	33 (38.8%)	17 (39.5%)
		Somewhat Agree, No Preference, or Somewhat Disagree	22 (25.9%)	10 (23.3%)
		Very Strongly Disagree, Strongly Disagree, or Disagree	30 (35.3%)	16 (37.2%)
			N=85	N=43
4	Would you be willing to pay a flat fee or a fee based on usage?	I would be willing to pay a flat fee	5 (5.9%)	1 (2.4%)
		I would be willing to pay a fee based on usage	19 (22.4%)	5 (11.9%)
		I would be willing to pay for either option	9 (10.6%)	4 (9.5%)
		I would not be willing to pay to use the tool	52 (61.2%)	32 (76.2%)
			N=85	N=42

**Table 3.** Journal Transparency Tool (JTT) Metric Preferences Voting Results.

#	Item	Round 1 ^a^		Round 2 ^a^		Round 3	
		Scale	n, %	Scale	n, %	Scale	%
1	A metric reporting whether the journal is indexed in PubMed or not	1–3	15 (17.4%)	1–3	6 (14.3%)	Include	47%
		4–6	14 (16.3%)	4–6	12 (28.6%)	Exclude	42%
		7–9	57 (66.3%)	7–9	24 (57.1%)	Abstain	11%
			N=86		N=42		
2	A metric reporting whether the journal is indexed in Scopus or not	1–3	15 (17.4%)	1–3	8 (19.0%)	Include	55%
		4–6	17 (19.8%)	4–6	9 (21.4%)	Exclude	35%
		7–9	54 (62.8%)	7–9	25 (59.5%)	Abstain	10%
			N=86		N=42		
3	A metric reporting whether the journal is indexed in Web of Science or not	1–3	13 (15.1%)	1–3	7 (16.7%)	Include	52%
		4–6	14 (16.3%)	4–6	12 (28.6%)	Exclude	33%
		7–9	59 (68.6%)	7–9	23 (54.8%)	Abstain	14%
			N=86		N=42		
4	A metric reporting whether the journal is a member of COPE or not	1–3	8 (9.3%)	1–3	4 (9.5%)	**Include**	**86%**
		4–6	14 (16.3%)	4–6	7 (16.7%)	Exclude	10%
		7–9	64 (74.4%)	7–9	31 (73.8%)	Abstain	5%
			N=86		N=42		
5	A metric reporting whether the journal is a member of CrossRef or not	1–3	9 (9.1%)	1–3	3 (7.1%)	Include	57%
		4–6	17 (13.6%)	4–6	7 (16.7%)	Exclude	33%
		7–9	59 (77.3%)	7–9	32 (76.2%)	Abstain	10%
			N=85		N=42		
6	A metric reporting whether the journal uses DOIs or not	1–3	8 (9.3%)	1–3	2 (4.8%)	*Consensus achieved in Round 2*	
		4–6	11 (12.8%)	4–6	3 (7.1%)		
		7–9	67 (77.9%)	**7–9**	**37 (88.1%)**		
			N=86		N=42		
7	For open access journals, a metric reporting whether the journal is listed in the DOAJ or not	1–3	6 (7.1%)	1–3	2 (4.8%)	**Include**	**81%**
		4–6	12 (14.1%)	4–6	9 (21.4%)	Exclude	5%
		7–9	67 (78.8%)	7–9	31 (73.8%)	Abstain	14%
			N=85		N=42		
8	A metric reporting whether the journal uses ORCIDs or not	1–3	11 (12.8%)	1–3	3 (7.1%)	**Include**	**86%**
		4–6	14 (16.3%)	4–6	8 (19%)	Exclude	10%
		7–9	61 (70.9%)	7–9	31 (73.8%)	Abstain	5%
			N=86		N=42		
9	A metric reporting whether the written content presented on the website is clear or not	1–3	10 (11.6%)	1–3	3 (7.1%)	Include	0%
		4–6	24 (27.9%)	4–6	11 (26.2%)	**Exclude**	**95%**
		7–9	52 (60.5%)	7–9	28 (66.7%)	Abstain	5%
			N=86		N=42		
10	A metric reporting whether the journal describes its approach to publication ethics or not	1–3	2 (2.3%)	*Consensus achieved in Round 1*			
		4–6	9 (10.5%)				
		**7–9**	**75 (87.2%)**				
			N=86				
11	A metric reporting whether the journal editors are listed or not	1–3	4 (4.7%)	*Consensus achieved in Round 1*			
		4–6	11 (12.8%)				
		**7–9**	**71 (82.6%)**				
			N=86				
12	A metric reporting whether the journal uses fake DOIs or not	1–3	4 (4.7%)	*Consensus achieved in Round 1*			
		4–6	9 (10.5%)				
		**7–9**	**73 (84.9%)**				
			N=86				
13	A metric reporting whether the journal reports misleading scholarly metrics or not	1–3	7 (8.2%)	*Consensus achieved in Round 1*			
		4–6	9 (10.6%)				
		**7–9**	**69 (81.2%)**				
			N=85				
14	A metric reporting whether a Transparency and Openness Practices (TOP) factor score is available or not	1–3	8 (9.6%)	1–3	4 (9.5%)	Include	17%
		4–6	28 (33.7%)	4–6	14 (33.3%)	Exclude	67%
		7–9	47 (56.6%)	7–9	24 (57.1%)	Abstain	17%
			N=83		N=42		
15	A metric reporting whether article peer reviews are openly reported or not	1–3	10 (11.8%)	1–3	5 (11.9%)	*Item modified for voting (See #16 &17)*	
		4–6	14 (16.5%)	4–6	12 (28.6%)		
		7–9	61 (71.8%)	7–9	25 (59.5%)		
			N=85		N=42		
16	A metric reporting whether all articles have an open access peer review	*Modified from #15*				Include	67%
						Exclude	28%
						Abstain	6%
17	A metric reporting whether peer reviewer names are disclosed for each article they reviewed	*Modified from #15*				Include	28%
						Exclude	44%
						Abstain	28%
18	A metric reporting whether there is verifiable contact information or not	1–3	3 (3.5%)	*Consensus achieved in Round 1*			
		4–6	8 (9.4%)				
		**7–9**	**74 (87.1%)**				
			N=85				
19	An option for the JTT to collect/share journal incidents	1–3	6 (7.1%)	1–3	1 (2.4%)	Include	12%
		4–6	29 (34.1%)	4–6	14 (33.3%)	**Exclude**	**81%**
		7–9	50 (58.8%)	7–9	27 (64.3%)	Abstain	6%
			N=85		N=42		
20	A metric reporting whether the journal has any article submission or processing fees and information about the journals funding model	*Item added based on Round 1 responses*		1–3	1 (3.0%)	*Item modified for voting (See #21 &22)*	
				4–6	7 (21.2%)		
				7–9	25 (75.8%)		
					N=33		
21	A metric reporting whether the journal has any article submission or processing fees	*Modified from #20*				**Include**	**94%**
						Exclude	6%
						Abstain	0%
22	A metric providing information about the journal’s funding model	*Modified from #20*				Include	76%
						Exclude	18%
						Abstain	6%
23	A metric reporting the journal’s citation metrics	*Item added based on Round 1 responses*		1–3	10 (30.3%)	Include	18%
				4–6	8 (24.2%)	Exclude	65%
				7–9	15 (45.5%)	Abstain	18%
					N=33		
24	A metric reporting the journal’s policies surrounding equity, diversity, and inclusion	*Item added based on Round 1 responses*		1–3	4 (12.1%)	Include	35%
				4–6	6 (18.2%)	Exclude	41%
				7–9	23 (69.7%)	Abstain	24%
					N=33		
25	A metric reporting the journal publisher’s size and how the JTT’s evaluation may negatively affect smaller publishers just by virtue of their size	*Item added based on Round 1 responses*		1–3	4 (12.1%)	Include	76%
				4–6	15 (45.5%)	Exclude	18%
				7–9	14 (42.4%)	Abstain	6%
				N=33			
26	A metric reporting the journal’s policies on retractions/corrections	*Item added based on Round 1 responses*		1–3	0 (0.0%)	*Consensus achieved in Round 2*	
				4–6	3 (9.1%)		
				7–9	**30 (90.9%)**		
					N=33		
27	A metric reporting the journal’s policies regarding reporting ethics, funding, and conflicts of interest	*Item added based on Round 1 responses*		1–3	0 (0.0%)	*Consensus achieved in Round 2*	
				4–6	1 (3.0%)		
				**7–9**	**32 (97.0%)**		
					N=33		
28	A metric reporting the journal’s peer review model and metrics	*Item added based on Round 1 responses*		1–3	0 (0.0%)	*Consensus achieved in Round 2*	
				4–6	3 (9.1%)		
				**7–9**	**30 (90.9%)**		
					N=33		
29	Should we move away from the Red/Green/orange colouration in the tool?	*Item added based on Round 3 discussion*				Yes	50%
						No	44%
						Abstain	6%
30	Should we aggregate all bibliographic database indexing in a single metric (PubMed, Scopus, Web of Science, etc.) in the JTT?	*Item added based on Round 3 discussion*				Yes	63%
						No	32%
						Abstain	5%

*Note*: Bolded numbers indicate consensus.

^a^ Round 1 and 2 items were scored on a 9-point scale, where 1 to 3 points were categorized as ‘unimportant,’ 4 to 7 points were categorized as ‘neutral,’ and 7 to 9 points were categorized as ‘important’ for inclusion within the tool.

### Round 1

**Participants**

A total of 86 participants completed the round 1 survey (see Table 1 for participant demographic characteristics). Respondents voted on JTT user feature ( Table 2) and JTT metric item ( Table 3) preferences and answered open-ended survey questions regarding the JTT. Please view the following link for complete round 1 responses: https://osf.io/92zh8.

Participants had less than 1 year (n = 1, 1.2%), 1 to 3 years (n = 10, 11.6%), 4 to 10 years (n = 34, 39.5%), 11 to 20 years (n = 21, 24.4%), and over 20 years (n = 20, 23.3%) of experience working in the publishing industry, with most being involved as an editor and/or with completing editorial tasks (n = 47, 55.3%). Editorial processes/peer review (n = 72, 81.8%), the publication process (n = 68, 77.3%), and open access (n = 64, 72.7%) were the top three areas of expertise in publishing that respondents identified with. Participants worked with over 70 international publishers/publishing organizations at the time of the study (see https://osf.io/92zh8 for the complete, unedited list of institutions).

**JTT user features**

Participants voted on four JTT user feature items in round 1 ( Table 2). None of the JTT user feature items reached the 80% consensus threshold after both rounds of voting. Participants provided the most support for: hosting the JTT on a website over an application programming interface (API)/browser plugin interface (n = 51, 58.0%); not paying to use the tool (n = 52, 61.2%); and fully automating the JTT (n = 41, 48.2%). The use of registration to access the JTT platform was contentious, with roughly equal proportions of participants being in support (n = 33, 38.8%), neutral (n = 22, 25.9%), and opposed to its use (n = 30, 35.3%). Open-survey responses regarding the JTT user format suggested that participants were predominantly concerned with ensuring that the JTT will be user-friendly and accessible.

**JTT metrics**

Of the 17 JTT metric items that participants voted on within round 1, five reached consensus ( Table 3). The following items were agreed upon as ‘important’ to include in the JTT on a 9-point scale: a metric that reports whether (1) the journal describes its approach to publication ethics or not; (2) the journal editors are listed or not; (3) the journal uses fake digital object identifiers (DOIs) or not; (4) the journal reports misleading scholarly metrics or not; and (5) there is verifiable contact information or not.

Participants suggested seven novel JTT metrics to vote on, including metrics that report: (1) whether the journal has any article submission or processing fees and information about the journal’s funding model; (2) the journal’s citation metrics; (3) the journal’s policies surrounding equity, diversity, and inclusion; (4) the journal’s policies on retractions/corrections; (5) the journal publisher’s size and how the JTT’s evaluation may negatively affect smaller publishers just by virtue of their size; (6) the journal’s policies regarding reporting ethics, funding, and conflicts of interest; and (7) the journal’s peer review model and metrics. All seven JTT metrics were added in for participants to vote for in round 2. Within open-ended survey responses, participants also raised concerns about how ‘transparency’ is defined by the tool and how metric scoring may negatively affect smaller publishers/journals.

### Round 2

**Participants**

Forty-three (50% of round 1) participants completed the round 2 survey. Respondents voted on JTT metric and user feature items that did not reach consensus in round 1, as well as any additional items suggested by participants in round 1 (see Table 3 notes for newly added items). Please view the following link for complete round 2 responses: https://osf.io/nh6cv.

Most respondents had more than 4 years of experience working in the publishing industry (n = 39, 90.7%). Similar to round 1, most participants were involved as an editor and/or completed editorial tasks (n = 26, 60.5%), and the top three areas of expertise within publishing participants identified were editorial processes/peer review (n = 38, 88.4%), the publication process (n = 35, 81.4%), and open access (n = 34, 79.1%). At the time of the study, participants in this round worked for 30 international publishers/publishing organizations (see https://osf.io/nh6cv for the complete, unedited list of institutions).

**JTT user features**

In round 2, participants voted on four JTT user features ( Table 2). While none of the items reached the 80% consensus threshold, respondents displayed similar voting preferences to round 1. Most participants supported hosting the JTT on a website over an API/browser plugin interface (n = 33, 76.7%), not paying to use the tool (n = 32, 76.2%), and fully automating the JTT (n = 24, 55.8%). Furthermore, similar to round 1, the use of registration to access the JTT platform was contentious, with roughly equal proportions of participants being in support (n = 17, 39.5%), neutral (n = 10, 23.3%), and opposed to (n = 16, 37.2%) its use. Open-survey responses regarding the JTT user format suggested that participants were predominantly concerned with ensuring that the JTT would be user-friendly, easy to understand, and concise.

**JTT metrics**

In round 2, participants voted on their preferences regarding 19 JTT metric items (12 metric items that did not reach consensus in round 1 and seven novel items added based on round 1 responses). Of these 19 items, four met the 80% consensus threshold. Participants found the inclusion of the following items to be ‘important’ on a 9-point scale: a metric reporting (1) the journal’s policies regarding reporting ethics, funding, and conflicts of interest; (2) the journal’s policies on retractions/corrections; (3) the journal’s peer review model and metrics; and (4) whether the journal uses DOIs or not ( Table 3).

In open-ended survey responses, participants suggested eight novel JTT metrics to include for voting (see https://osf.io/nh6cv for the complete list). These new items were not voted on within round 3 due to time and resource constraints. Participants also suggested that the JTT metric scoring strategy should be openly shared to provide transparency and allow users to understand how metrics are evaluated in the tool’s assessment process. Furthermore, similar to round 1, participants raised concerns with how ‘transparency’ is defined. They added that the JTT should adopt a widely recognized definition of ‘transparency’ to ensure a clear understanding of the concept and its application within the tool.

### Round 3

**Participants**

In round 3, 21 participants discussed and voted on JTT metric items that did not reach consensus after round 2 ( Table 3) within an online consensus group meeting. Specifically, round 3 participants included EC, IC, PEC, JG, RBH, BSK, AM, EP, AP, JDBS, PRS, PS, JW, MW, and seven other publishers. Demographic characteristics were not collected for round 3 participants.

**JTT user features**

In round 3, JTT user feature items that did not meet consensus in round 2 were not discussed nor voted on due to time and resource constraints.

**JTT metrics**

During round 3, participants discussed and voted on the inclusion of JTT metrics within an online group meeting. There were 15 items that had not reached consensus in round 2. After discussing each item, two individual items were modified to be expanded into two items each, resulting in four items in total, and two novel items were added for voting (see notes on modifications made in Table 3). With these revisions, participants ultimately voted on 19 JTT metric items in round 3.

Of the 19 JTT metric items voted on in round 3, six met the 80% consensus threshold. Participants supported the *inclusion* of four items: a metric (1) reporting whether the journal is a member of COPE or not; (2) reporting whether the journal has any article submission or processing fees; (3) reporting whether the journal uses ORCIDs or not; and (4) reporting whether the journal is listed in the DOAJ or not. Participants supported the *exclusion* of two items: (1) an option for the JTT to collect/share journal incidents; and (2) a metric reporting whether the written content presented on the website is clear or not. In total, 15 out of the 30 JTT metrics reached consensus after all three rounds of voting (see Table 4 for the complete list of consensus items).

**Table 4.** Delphi items that reached consensus after all three rounds of voting.

#	Item	Round consensus was reached	Score/Decision ^a^	n (%)
1	A metric reporting whether the journal describes its approach to publication ethics or not	1	Important (7–9)	75 (87.2%)
2	A metric reporting whether there is verifiable contact information or not	1	Important (7–9)	74 (87.1%)
3	A metric reporting whether the journal uses fake DOIs or not	1	Important (7–9)	73 (84.9%)
4	A metric reporting whether the journal editors are listed or not	1	Important (7–9)	71 (82.6%)
5	A metric reporting whether the journal reports misleading scholarly metrics or not	1	Important (7–9)	69 (81.2%)
6	A metric reporting the journal’s policies regarding reporting ethics, funding, and conflicts of interest	2	Important (7–9)	32 (97.0%)
7	A metric reporting the journal’s policies on retractions/corrections	2	Important (7–9)	30 (90.9%)
8	A metric reporting the journal’s peer review model and metrics	2	Important (7–9)	30 (90.9%)
9	A metric reporting whether the journal uses DOIs or not	2	Important (7–9)	37 (88.1%)
10	A metric reporting whether the journal has any article submission or processing fees	3	Include	94%
11	A metric reporting whether the journal is a member of COPE or not	3	Include	86%
12	A metric reporting whether the journal uses ORCIDs or not	3	Include	86%
13	For open access journals, a metric reporting whether the journal is listed in the DOAJ or not	3	Include	81%
14	A metric reporting whether the written content presented on the website is clear or not	3	Exclude	95%
15	An option for the journal transparency tool to collect/share journal incidents	3	Exclude	81%

^a^ Round 1 and 2 items were scored on a 9-point scale, where 1 to 3 points were categorized as ‘unimportant,’ 4 to 7 points were categorized as ‘neutral,’ and 7 to 9 points were categorized as ‘important’ for inclusion within the tool.

A thematic content analysis ^18^ of the online group meeting transcripts resulted in two themes (see https://osf.io/978vs for the thematic analysis and Table 5 for a summary of the themes). First, several factors influenced the support individuals had for JTT metrics. This included how relevant the metric was to transparency and open practices; if the information gathered by the metric was already captured within another one; and how challenging it would be to gain membership for that metric when applicable (e.g., to be indexed on DOAJ). Concerns were raised within multiple rounds regarding how certain metrics could be exclusionary towards journals (e.g., small publishers) based on selection criteria that are not related to adherence to transparency and open practices (e.g., language, costs, geographic location). For items that reached consensus for exclusion in round 3, the primary issue raised by participants was the inability to objectively define and evaluate the relevant metrics using a clear criterion on the JTT. Within the second theme, participants provided suggestions to increase user clarity. Respondents stated that, with each JTT metric, users should be provided clear descriptions of what the metric is, how it is measured on the JTT, and potential biases that may be associated with the metric. Further, participants suggested that the naming of the JTT and its components should be clear and accurate. Some respondents stated that the tool should be renamed (i.e., the tool should not be called the ‘Journal Transparency Tool’) to increase clarity, but alternative names were not proposed.

**Table 5.** Round 3 Delphi Online Consensus Group Themes.

Themes	Codes	Code description	Example quotes ^a^
Factors impacting support for journal transparency tool (JTT) Metrics	Metric Scoring System	Participants provided support for metrics based on if there was an objective means and clear criteria to score the metric.	“How is this going to be assessed?” (P1)
	Transparency	Participants provided support for metrics based on how relevant they are to transparency and open practices.	“Looking at transparency, I do think it is relevant…” (P2)
	Redundancy	Participants provided support for metrics based on if the information gathered by the metric also captured in another metric.	“ … if it’s not going to be included already…” (P3)
	Membership Criteria	Participants provided support for metrics based on how challenging it is to gain membership for that metric (e.g., to be indexed on DOAJ, COPE), when relevant.	“…membership…takes a very long time.” (P1)
	Exclusionary selection criteria	Participants provided support for metrics based on how biased a metric may be against journals (e.g., small publishers) based on selection criteria that is not related to transparency and open practices (e.g., language, costs, location).	“ … concern is… the cost for journals in lower-middle income countries” (P4)
Suggestions for User Clarity	Metric Descriptions	Users should be given descriptions of what the metric is, how it is measured on the JTT, and potential biases associated with the metric.	“…to have a more general disclaimer … ” (P2)
	Naming Labels	The naming of the JTT and its components should be clear and accurate.	“We’re talking a lot about metrics here, but some of these are more like indicators.” (P5)

^a^ P refers to participant ID.

## Discussion

The aim of this three-round Delphi study was to determine the needs and preferences of the publishing community for a JTT. A total of 86 and 43 participants completed an online survey in rounds 1 and 2, respectively (see Table 1 for demographic information). In round 3, 21 participants discussed and voted on JTT items that did not reach consensus after round 2 within an online consensus group meeting. After all rounds of voting, none of the four JTT user feature item preferences obtained consensus ( Table 2). However, 15 out of a total of 30 JTT metric items ( Table 3) reached the 80% consensus threshold (see Table 4 for the full list of consensus items). An analysis of the round 3 online group transcript resulted in two themes: ‘factors impacting support for JTT metrics’ and ‘suggestions for user clarity’ ( Table 5).

Ensuring the tool will be accessible and easy-to-use appeared to be the primary priority for participants. Several suggestions were provided to ensure that the tool has these properties. First, participants suggested that JTT metrics should have clear descriptions of what the metric is and what it is intended to measure. Further, while none of the JTT user features reached consensus, respondents within open survey questions emphasized that user features (e.g., the tool’s browser interface, the use of registration to access the tool) should be accessible through a reliable and user-friendly platform and that information on the tool should be presented concisely in easily digestible formats. Finally, participants suggested that the naming of the tool itself and tool components should be clear and accurate. All these suggestions will be considered during the implementation of the tool.

Participants also appeared to be concerned about the tool’s adherence to transparency. Specifically, with there being multiple ways to conceptualize transparency and open practices within biomedical research, ^19^^–^^21^ participants raised concerns with how ‘transparency’ would be defined and implemented on the JTT. In addition, respondents suggested that the JTT scoring strategy should be shared openly to increase transparency and allow users to understand how metrics are weighted/evaluated in the tool’s assessment process. In response to this feedback, we will adopt a widely recognized definition of ‘transparency’ on the JTT to ensure a clear understanding of the concept and its application within the tool. Similarly, the JTT scoring system for metric evaluation will be readily available for users to access and understand on the tool itself.

Concerns that the JTT may increase inequity within the international scientific community were also raised by several participants. Specifically, respondents in all rounds indicated that certain publishers (e.g., publishers from low-income countries, and non-English language journals) may not be able to obtain ‘high’ scores on JTT metrics due to barriers related to costs, language, and/or geographic location. These journals, which already face barriers to engagement, ^22^^,^^23^ may consequently be penalized due to the JTT scoring system and lose readership despite not engaging in suboptimal transparency and open practices. To respond to these concerns, we will actively work on mitigating biases associated with the tool. This may include, but is not limited to, translating sections of the JTT to reduce language barriers and/or adding informative descriptive labels to notify users about biases associated with JTT metrics. Community stakeholders will also be asked about potential biases associated with the JTT’s scoring system during iterative consultations throughout the timeline of the tool’s development.

This study allowed us to establish publishing community preferences for the JTT and represents one study of a three-part initiative to determine the needs and preferences of stakeholder groups (i.e., patients, ^4^ researchers/clinicians, ^3^ and publishers) for the JTT. Considering the needs of the publishing community in our design of the JTT will help to ensure the tool we develop resonates with the publisher community and fulfills needs that they identify. The next phase of development will require integrating preferences from all stakeholder groups and developing the tool for use. Ultimately, some items that reached consensus may not be possible to include depending on how preferences between different stakeholders correspond with each other and decisions the team will make regarding user features (e.g., if we fully automate the tool, some metrics may not lend well to automation). We anticipate iterative consultation with the community as we work to develop the JTT that best meets users’ needs.

This study has several strengths. First, the participant sample included a heterogeneous and diverse group of publishers that represented over 70 international institutions (see https://osf.io/nh6cv for the complete list of institutions). Further, the use of an open invitation to participate for all publishers allowed us to reduce the potential for bias that would come if we had approached individual publishers and/or used a single sampling approach. Another strength of this Delphi study is the use of a cross-sectional survey and an online consensus group to determine participant preferences. While the survey identified broad trends in journal preferences, the online consensus group allowed us to determine the specific motivations behind why participants provided support or opposition to survey items. Conversely, one weakness of the present analysis is that we only included participants fluent in the English language, so our findings may not be representative of individuals who do not publish in English. ^24^

## Conclusion

This study aimed to identify the preferences of the publishing community for a JTT. We conducted a three-round modified Delphi with publishers that were recruited through purposeful and snowball sampling. The first two Delphi rounds involved an online survey, and the third round occurred through an online consensus group discussion. After all rounds of voting, fifteen out of 30 JTT metric items, but none of the four JTT user feature items reached consensus. Participants suggested that their primary concerns for a JTT are to ensure that it is relevant, user-friendly, accessible, and equitable. Results from this analysis will be used to inform the development of the JTT to help enable and sustain robust transparency and open practices within the scientific community.

## Ethical considerations

Research ethics approval was obtained from the Ottawa Health Science Network Research Ethics Board (OSHN-REB#: 20220132-01H) on April 06, 2022. The final protocol was registered on the Open Science Framework (OSF) and can be found at https://osf.io/ur67d. Participants provided implied consent to participate during three rounds of the study, as approved by the aforementioned REB with this study being deemed as having minimal risk. This study was conducted in adherence with the Declaration of Helsinki.

## Data Availability

Open Science Framework: Publisher Preferences for a Journal Transparency Tool: A Delphi Study, https://doi.org/10.17605/OSF.IO/8V63X. ^25^ Data are available under the terms of the Creative Commons Attribution 4.0 International license (CC-BY 4.0). We followed the ACcurate COnsensus Reporting Document (ACCORD) checklist in reporting our findings.
