# Supplementary material for: Publisher preferences for a journal transparency tool: A modified three-round Delphi study
Source: F1000Res. 2025 Jan 31;13:915. Originally published 2024 Aug 9. [Version 2] doi: 10.12688/f1000research.154408.2 (PMC11880752; doi:10.12688/f1000research.154408.2)
Supplement: Peer review history

# Reviewer report: Reviewer response for version 2

**DOI:** 10.5256/f1000research.174029.r364197

**Published:** 2025-03-04

**Authors:** Shubhada Nagarkar

**Affiliations:** 1 Department of Library and Information Science and Centre for Publication Ethics, Savitribai Phule Pune University, Pune, Maharashtra, India

Summary

 A tool to check predatory of status of journals is very much required in this age and this paper deals with that need.

 This paper checks feasibility and utility value of a tool (JTT) that is proposed to check predatory status of the journal.  To conduct this survey, a Delphi method (three rounds) is used with elaborate questionnaire. A detailed analysis has been carried out to support the conclusion.

 Is the work clearly and accurately presented and does it cite the current literature?

 - What is JTT tool is not explained in the initial part of the paper. The background, objectives and requirements of JTT are not clearly elaborated.

 - References are properly cited.

 Suggestions

 The purpose of JTT should be elaborated. It would be helpful to include a prototype of  JTT to help the audience visualize JTT and its functionality.

Is the work clearly and accurately presented and does it cite the current literature?

Partly

If applicable, is the statistical analysis and its interpretation appropriate?

Yes

Are all the source data underlying the results available to ensure full reproducibility?

Yes

Is the study design appropriate and is the work technically sound?

Yes

Are the conclusions drawn adequately supported by the results?

Yes

Are sufficient details of methods and analysis provided to allow replication by others?

Yes

Reviewer Expertise:

publication ethics

I confirm that I have read this submission and believe that I have an appropriate level of expertise to confirm that it is of an acceptable scientific standard, however I have significant reservations, as outlined above.

# Reviewer report: Reviewer response for version 2

**DOI:** 10.5256/f1000research.174029.r363719

**Published:** 2025-02-17

**Authors:** Andreas Nishikawa-Pacher

**Affiliations:** 1 Bibliothek, Technische Universitat Wien (Ringgold ID: 27259), Vienna, Austria

We are now at version 2 of this article; with regards to the former version, my two main concerns had related to (1) the sharing of survey data, and (2) a lack of engagement with similar projects and relevant meta-scientific literature. 

 The authors addressed the first aspect by uploading survey data to OSF [1]. The data there indeed seem comprehensive (though not machine-readable), and scrolling around the responses renders visible the strengths of the survey (which the authors have duly underlined in their paper). The numerous comments from the respondents, coupled with the plurality of institutions and roles they represent, provide an invaluable source into how editorial staff members, researchers, and university administrators approach the issue. It is natural that the paper itself cannot report on every single statement, so reading about some specific desires from the respondents was interesting.

 Examples include a wished-for integration with OJS, or the wish for a built-in appraisal system, as stated in p. 22 of https://osf.io/nh6cv; and listing metrics like whether a journal uses plagiarism checks, or whether a journal publishes corrigenda, whether a journal is run by a scientific society, or whether rejection rates are named, or whether the journal cooperates with archival services like CLOCKSS, whether the journal published open abstracts [I4OA], or the age of a journal, as listed in p. 25 of that document. In other words, the responses contain a huge wealth of ideas that can be tapped for later use (even if not necessarily for the JTT itself). The sharing of the survey dataset is thus very valuable.

 At the same time, one can see various critical comments in the comments from the respondents. It would have been interesting to see how the authors addressed the most critical of them. (Just to quote some exemplary snippets: “It looks like your tool will serve the already heavily monopolized publishing industry”; “It is still unclear what exactly this tool is trying to do”; “It is unclear what the purpose of the journal transparency tool is given that many of the measures don't relate to transparency, and they would not assess whether a journal is predatory. Who is the intended audience and what do you expect them to learn from the tool that they can't learn elsewhere?”).

 The second issue I had raised – a closer engagement with similar initiatives and the relevant meta-scientific literature – was dealt with, in my view and if I did not overlook anything, only superficially via the addition of quick references *en passant*, but the authors made clear that they are still at a preliminary stage so that a full-fledged comparison is not yet possible.

 Acknowledging that the authors are still in an early process, and that they have already done a lot of survey-related work on this matter, I would not insist on this point any longer.

 Finally, I had raised some other issues (such as the content of the 'Results' section in the abstract, or a reflection on defining ‘transparency’ in this context) that the authors engaged with very well. I would thus not ask for any more changes to this manuscript, given the specific context of the project’s preliminary stage, and I am excited to see where the JTT heads to.

 (As a final remark, I would perhaps only hint to how OpenAlex does contain relevant data on a journal [or rather: source] level, which may also merit a closer look at a later stage: https://docs.openalex.org/api-entities/sources)

Is the work clearly and accurately presented and does it cite the current literature?

Partly

If applicable, is the statistical analysis and its interpretation appropriate?

Yes

Are all the source data underlying the results available to ensure full reproducibility?

Partly

Is the study design appropriate and is the work technically sound?

Yes

Are the conclusions drawn adequately supported by the results?

Yes

Are sufficient details of methods and analysis provided to allow replication by others?

Yes

Reviewer Expertise:

meta-science

I confirm that I have read this submission and believe that I have an appropriate level of expertise to confirm that it is of an acceptable scientific standard.

# Reviewer report: Reviewer response for version 2

**DOI:** 10.5256/f1000research.174029.r363720

**Published:** 2025-02-07

**Authors:** Bahar Memarian

**Affiliations:** 1 Simon Fraser University, Burnaby, British Columbia, Canada

It would have been better if the authors had kindly provided a point-by-point summary of changes for each of the comments before full indexing. A point-by-point response and identification of where it had been addressed in the document would be helpful.

Is the work clearly and accurately presented and does it cite the current literature?

Partly

If applicable, is the statistical analysis and its interpretation appropriate?

I cannot comment. A qualified statistician is required.

Are all the source data underlying the results available to ensure full reproducibility?

Partly

Is the study design appropriate and is the work technically sound?

No

Are the conclusions drawn adequately supported by the results?

Partly

Are sufficient details of methods and analysis provided to allow replication by others?

Partly

Reviewer Expertise:

The role of AI in higher education research, higher education research, engineering education research, human factors engineering

I confirm that I have read this submission and believe that I have an appropriate level of expertise to confirm that it is of an acceptable scientific standard, however I have significant reservations, as outlined above.

# Reviewer report: Reviewer response for version 1

**DOI:** 10.5256/f1000research.169431.r320276

**Published:** 2024-09-13

**Authors:** Bahar Memarian

**Affiliations:** 1 Simon Fraser University, Burnaby, British Columbia, Canada

Thank you for your work. Please find suggestions on areas of improvement below:

  

- It is unclear what the tool has to offer beside curating and collecting information that is available off the web
- The research questions and goal is missing and the work presented is more of an organizational effort as opposed to a research (e.g., review or empirical paper with clear gap, purpose and methodology)
- The selection and rationale for survey design is not clear
- The outcomes of the study does not add to new knowledge formation
- Overall the study needs to be revisited and a clear research goal and study design needs to be considered. Given the large number of authors, the contribution and relevance of their work to this topic is also unjustified

Is the work clearly and accurately presented and does it cite the current literature?

Partly

If applicable, is the statistical analysis and its interpretation appropriate?

I cannot comment. A qualified statistician is required.

Are all the source data underlying the results available to ensure full reproducibility?

Partly

Is the study design appropriate and is the work technically sound?

No

Are the conclusions drawn adequately supported by the results?

Partly

Are sufficient details of methods and analysis provided to allow replication by others?

Partly

Reviewer Expertise:

The role of AI in higher education research, higher education research, engineering education research, human factors engineering

I confirm that I have read this submission and believe that I have an appropriate level of expertise to confirm that it is of an acceptable scientific standard, however I have significant reservations, as outlined above.

# Response

**Published:** 2024-10-28

**Authors:** Jeremy Ng

**Affiliations:** University of Ottawa, Canada

We kindly thank the reviewer for providing their feedback on our manuscript.

 Regarding the research goals, we envision the JTT as a tool that allows stakeholders to more easily determine a journal’s operations and transparency practices, which will include publicly available information. This could especially be helpful for prospective tool users that are unfamiliar with what constitutes less transparent practices and are unsure where online to look for such information. For instance, a survey of medical oncologists found that 45.2% of participants indicated a lack of knowledge in identifying journals with predatory backgrounds [1]. Other work on researchers publishing in presumed predatory journals had found 28.3% of participants being previously unaware of the potentially predatory nature of the journal they had published in [2]. We have described the prospective value of the tool in the Introduction:

 “A JTT, as we envision it, could be an automated tool to provide users with information about a given journal’s transparency practices. This in turn could help users decide on the journal’s credibility and whether to interact with it (e.g., read and cite articles published in it, submit manuscripts to it). The tool is expected to add value by providing stakeholders with information about how a journal operates and which best practices it meets. The benefit of this tool may be most obvious for producers of research looking to publish, as well as readers of research looking to consume evidence to help inform healthcare-related decisions. Additionally, this tool may be of great value to new researchers that are being introduced to the research community and individuals in the profession of evaluating, publishing, and indexing research.”

 In terms of the survey design, this research is one of a three-part initiative to obtain preferences for a journal transparency tool from stakeholders of patients, researchers and clinicians, and publishers. A modified Delphi methodology was conducted to obtain publishers’ preferences in particular, which we believe is beneficial in understanding how to serve the needs of the greater scientific community. The last paragraph of the introduction describes and elaborates on our decision to utilize this methodology:

 “The Delphi method is a means of structuring communication between experts around a complex issue to make a collective judgment on a given topic. It is a social science technique but has been applied in other settings, including healthcare environments (see for example: [5,6]). The Delphi approach allows for effective communication by individual group members and the group as a whole [7]. It limits direct confrontation between individuals, fosters independent thought by the experts involved, and aims to achieve consensus within a group without the need for a leader [8]. By using the Delphi method, we established the international English-speaking publisher communities’ consensus view on what should be included in a JTT, which will contribute to meaningfully situating the tool within the scholarly landscape and help to ensure that the most relevant inputs are used to build the tool.”

 The contributions of each author have been enumerated in the author details, in accordance with CRediT (Contributor Role Taxonomy) roles. The contributions of each author have been significant and relevant to this work and justify authorship.

1. Richtig G, Richtig E, Böhm A, Oing C, Bozorgmehr F, Kruger S, Kiesewetter B, Zielinski C, Berghoff AS. Awareness of predatory journals and open access among medical oncologists: results of an online survey. ESMO open. 2019 Jan 1;4(6):e000580.
2. Cobey KD, Grudniewicz A, Lalu MM, Rice DB, Raffoul H, Moher D. Knowledge and motivations of researchers publishing in presumed predatory journals: a survey. BMJ open. 2019 Mar 1;9(3):e026516.

**Response to Reviewers Summary**

 We thank both reviewers for their feedback. In response to comments provided by Andreas Nishikawa-Pacher, machine-readable survey results have been uploaded to Open Science Framework (OSF), and references to similar initiatives (e.g., the Journal Observatory) were added to the manuscript. Further, concerns raised by this reviewer regarding defining 'transparency' and difficulties with feasibly enacting all participant preferences are acknowledged and appreciated. In response, we note that the Journal Transparency Tool (JTT) is currently within preliminary stages, and defining such items will require the collation of data from multiple stakeholders, leading to the limited information being provided regarding such elements within the present report. Further, we appreciate the comment that the abstract’s results may be more interesting if it focused on the contents of the survey outcome rather than numerical data. However, we believe that the present focus aptly represents this report’s findings due to the ‘numeric’ nature of the data provided by the present study design (i.e., a modified Delphi). Alternatively, we appreciate the concerns raised by Bahar Memarian regarding the tool's contribution and design, along with the purpose of the present research study. In response, we clarify that the JTT aims to help users identify journals that adhere to transparent research practices and highlight how this addresses a known gap within the literature. Further, the goal of this research study was to use a modified Delphi approach to capture publisher preferences for the JTT. The sections of the manuscript highlighting such information have been provided within our response for further clarity.

# Reviewer report: Reviewer response for version 1

**DOI:** 10.5256/f1000research.169431.r313617

**Published:** 2024-08-27

**Authors:** Andreas Nishikawa-Pacher

**Affiliations:** 1 Bibliothek, Technische Universitat Wien (Ringgold ID: 27259), Vienna, Austria

Thank you for inviting me to review this document. It reports about a survey of publishers' wishes regarding what a 'journal transparency tool' should entail. The document describes the three-round Delphi survey in minute detail; it all seems plausible and honest, and the whole project seems to aim for a platform that will be useful for many scholars, including meta-scientists. (I personally would be delighted if there was a reliable, up-to-date platform that would tell me which journals publish registered reports!)

 There is not much to criticize. the one thing that seemed missing was the availability of machine-readable, structured data of the survey results (e.g., in a CSV or Excel format), given that one of the pre-formulated questions of this peer review report is whether "all the source data underlying the results available to ensure full reproducibility". In addition, I think the abstract's "Results" section would be more interesting if it was not mainly about numbers, but rather about the contents of the survey outcome. Finally, in the final section, announcing a definition of 'transparency' that the authors plan to use would have been interesting as well (rather than simply to state that some kind of agreeable definition will be used).

 Other than that, there is only one aspect that I would raise. Namely, the whole report seems to mainly revolve around the authors' very own project. I would have expected, especially in the Introduction and the Discussion, a much more broader 'horizontal' view with regards to similar efforts that may be or may have been underway. 

 For instance, there used to be the "Journal Observatory" from a mainly Dutch team at https://www.journalobservatory.org/prototype/ -- or the "Platform for Responsible Editorial Policies" (PREP [2]) and related initiatives (in fact, the latter's website has a "related initiatives" section at https://www.responsiblejournals.org/relatedinitiatives). What are the project's similarities and differences with regards to that? There are also less comprehensive and more granular, single-issue-focused efforts of analyzing scholarly journals which may merit a discussion. They may address something highly specific, such as a call for journals to harmonize reporting standards of radiation dosimetry [1], or they may be more general, such as my dataset on journals' Twitter accounts [3] . Is it intended to do a survey of such all-discipline-encompassing journal-level initiatives to see which of them can be integrated into such a JTT tool?

 Speaking of which -- by "which" I refer only partly to my self-citation (though another will come soon), instead I rather mean the "bottom-up" data collection undertaken by researchers external to the publishing industry -- the fact that this project may seem to operate automatically makes me wonder to what extent it is possible to enact such an automation with regards to many of the indicators, such as the use of fake DOIs, the misleading metrics, the open peer reviews, the availability of verifiable contact information, or the information about the journals' funding model. Is not the majority of the indicators listed in Table 3 impossible to fetch with full automation? This observation does prompt me to ask whether the project expects publishers themselves to provide the data to the JTT, or whether the information are to be scraped in a different way (and this is what leads me to the next self-citation that the beginning of this paragraph anticipated, namely my "Open Editors" project which used webscraping to collect data about editors from thousands of journals [4]). Can one reasonably expect publishers themselves to provide data in a standardized way? With my Open Editors project, I thought that the answer may be no... at least for now. It's too much of a huge coordination issue. With 20+ indicators other than the listing of editors, the problem is even multiplied. And if one believes that publishers themselves will be so observant & virtuous to provide all the data that will be asked from them, then what would be the additional costs imposed upon the companies to do that on a regular basis, and who would ultimately pay for that?

 These are just some practical aspects that I would think of which merits some discussion, I believe. They are not central to the actual Delphi survey, but they would 'frame' the report by differentiating itself from relevant other projects, by showing what similar initiatives exist, and by weighing the pros and cons in a more in-depth manner.

 Other than that, I believe the project offers a laudable goal, and it will be interesting to witness its further steps.

Is the work clearly and accurately presented and does it cite the current literature?

Partly

If applicable, is the statistical analysis and its interpretation appropriate?

Yes

Are all the source data underlying the results available to ensure full reproducibility?

Partly

Is the study design appropriate and is the work technically sound?

Yes

Are the conclusions drawn adequately supported by the results?

Yes

Are sufficient details of methods and analysis provided to allow replication by others?

Yes

Reviewer Expertise:

meta-science

I confirm that I have read this submission and believe that I have an appropriate level of expertise to confirm that it is of an acceptable scientific standard, however I have significant reservations, as outlined above.

## References

[1] : Recommendations for harmonized reporting of radiation Dosimetry by adoption of Compatibility in Irradiation Research Protocols Expert Roundtable (CIRPER). *Int J Radiat Biol* .2024;100(6) : 10.1080/09553002.2024.2331130 821-823 10.1080/09553002.2024.2331130 38568854

[2] : The Platform for Responsible Editorial Policies: An initiative to foster editorial transparency in scholarly publishing. *Learned Publishing* .2020;33(3) : 10.1002/leap.1312 340-344 10.1002/leap.1312

[3] : The Twitter accounts of scientific journals: a dataset. *Insights the UKSG journal* .2023;36: 10.1629/uksg.593 10.1629/uksg.593

[4] : Open Editors: A dataset of scholarly journals’ editorial board positions. *Research Evaluation* .2023;32(2) : 10.1093/reseval/rvac037 228-243 10.1093/reseval/rvac037

# Response

**Published:** 2024-10-28

**Authors:** Jeremy Ng

**Affiliations:** University of Ottawa, Canada

We kindly thank the reviewer for providing their feedback on our manuscript.

 The results section of the abstract principally focused on percentages of items that reached consensus due to this Delphi study’s purpose and design, which aimed to determine publisher preferences on the Journal Transparency Tool (JTT). As with most survey questions being quantitative in nature, we believe these numerical findings to be the most relevant study results. 

 With regards to declaring a definition of ‘transparency’, it was noted within the results that participants had raised concerns over how transparency would be defined, and we acknowledge that transparency in research can encompass different dimensions of research practices. As the studies identifying preferences for the development of the JTT involves three stakeholder groups of publishers, researchers and clinicians, and patients, we believe there is merit in determining a definition of transparency for the JTT on a later date, based on findings of all stakeholder groups. Consequently, a definition of transparency will not be included in this report.

 With regards to the survey results in a machine-readable form, we have now re-posted the publicly available results on OSF in an Excel format: https://osf.io/z546q

 However, the full underlying source data unfortunately cannot be shared due to REB restrictions.

 On the initiatives described by the reviewer, we have mentioned the Platform for Responsible Editorial Policies (PREP) and of the “Journal Observatory” to the Discussion section, “As a whole, similar to other initiatives [24,25], we believe that this tool will be beneficial in responding to calls for academic journals to become more transparent [1,2].” It was, however, not possible to contrast similarities and differences between these tools, as the JTT does not exist yet. Further, the present paper is focused on the preferences of journal publishers as a stakeholder for the planned JTT. We are not planning to conduct a survey into all journal-level initiatives.

 Further on the point of implementation, we acknowledge that there may be difficulties in implementing all participants’ preferences with regards to automation, as noted in the Discussion section (e.g., how some metrics may not lend well to automation, how the majority of participants would not pay for a tool). All three stakeholders’ preferences inform the creation of the JTT, but not all aspects may ultimately be feasibly implemented. However, this study lays the groundwork for the JTT’s future development.

**Response to Reviewers Summary**

 We thank both reviewers for their feedback. In response to comments provided by Andreas Nishikawa-Pacher, machine-readable survey results have been uploaded to Open Science Framework (OSF), and references to similar initiatives (e.g., the Journal Observatory) were added to the manuscript. Further, concerns raised by this reviewer regarding defining 'transparency' and difficulties with feasibly enacting all participant preferences are acknowledged and appreciated. In response, we note that the Journal Transparency Tool (JTT) is currently within preliminary stages, and defining such items will require the collation of data from multiple stakeholders, leading to the limited information being provided regarding such elements within the present report. Further, we appreciate the comment that the abstract’s results may be more interesting if it focused on the contents of the survey outcome rather than numerical data. However, we believe that the present focus aptly represents this report’s findings due to the ‘numeric’ nature of the data provided by the present study design (i.e., a modified Delphi). Alternatively, we appreciate the concerns raised by Bahar Memarian regarding the tool's contribution and design, along with the purpose of the present research study. In response, we clarify that the JTT aims to help users identify journals that adhere to transparent research practices and highlight how this addresses a known gap within the literature. Further, the goal of this research study was to use a modified Delphi approach to capture publisher preferences for the JTT. The sections of the manuscript highlighting such information have been provided within our response for further clarity.
